# Apixaban for treatment of confirmed heparin-induced thrombocytopenia: a case report and review of literature

**DOI:** 10.1186/s40164-017-0080-7

**Published:** 2017-07-14

**Authors:** Daniel E. Ezekwudo, Rebecca Chacko, Bolanle Gbadamosi, Syeda Batool, Sussana Gaikazian, Theodore E. Warkentin, Jo-Ann I. Sheppard, Ishmael Jaiyesimi

**Affiliations:** 1 Department of Hematology and Oncology, William Beaumont Hospital, Oakland University William Beaumont School of Medicine, 3577 W. 13 Mile Rd., Suite 202a, Royal Oak, MI USA; 2 Department of Internal Medicine, William Beaumont Hospital, Oakland University William Beaumont School of Medicine, Royal Oak, MI USA; 3 Department of Medicine, McMaster University, 1280 Main St W, Hamilton, ON L8S 4L8 Canada; 4 Department of Pathology & Molecular Medicine, McMaster University, 1280 Main St W, Hamilton, ON L8S 4L8 Canada

**Keywords:** Heparin-induced thrombocytopenia, Serotonin-release assay, Platelet factor 4, Direct oral anticoagulants, Argatroban, Apixaban, Case report

## Abstract

**Background:**

Heparin-induced thrombocytopenia (HIT) is a life and limb-threatening condition caused by the binding of platelet-activating antibodies (IgG) to multimolecular platelet factor 4 (PF4)/heparin complexes because of heparin exposure. The by-product of this interaction is thrombin formation which substantially increases the risk of venous and/or arterial thromboembolism. Currently, only one anticoagulant, argatroban, is United States Food and Drug Administration-approved for management of HIT; however, this agent is expensive and can only be given by intravenous infusion. Recently, several retrospective case-series, case reports, and one prospective study suggest that direct oral anticoagulants (DOACs) are also efficacious for treating HIT. We further review the literature regarding current diagnosis and clinical management of HIT.

**Case presentation:**

A 66-year-old male patient developed HIT beginning on day 5 post-cardiovascular surgery; the platelet count nadir on day 10 measured 16 × 10^9^/L. Both the PF4-dependent ELISA and Serotonin-release assay were strongly positive. Despite initial anticoagulation with argatroban (day 6), the patient developed symptomatic Doppler ultrasound-documented bilateral lower extremity deep vein thrombosis on day 14 post-surgery. The patient was transitioned to the DOAC, apixaban, while still thrombocytopenic (platelet count 108) and discharged to home, with platelet count recovery and no further thrombosis at 3-month follow-up.

**Conclusions:**

We report a patient with serologically confirmed HIT who developed symptomatic bilateral lower limb deep vein thrombosis despite anticoagulation with argatroban. The patient was switched to oral apixaban and made a complete recovery. Our patient case adds to the emerging literature suggesting that DOAC therapy is safe and efficacious for management of proven HIT.

## Background

Heparin-induced thrombocytopenia (HIT) results in high rate of thrombosis (>50%) and mortality (10–30%) if untreated [1, 2]. Current guidelines recommend cessation of heparin for patients strongly suspected of having HIT and prompt initiation of a non-heparin direct thrombin inhibitor for anticoagulation, such as argatroban or bivalirudin. These drugs are administered intravenously as continuous titratable infusions requiring frequent laboratory monitoring and dose adjustment, and are expensive. Given the high costs and prolonged hospitalization required for intravenously administered direct thrombin inhibitors, there have been efforts to seek alternatives that are cost effective and similarly efficacious [3]. Fondaparinux, an antithrombin-dependent selective factor Xa-inhibitor, has been shown to be a safe and effective alternative. The agent is provided as a daily subcutaneous injection and does not require routine laboratory monitoring; thus, its relatively low drug cost and potential clinical utility makes it an attractive choice [4]. Nevertheless, fondaparinux is given by subcutaneous injection, which makes post-discharge administration problematic or undesirable for many patients. Recently, intriguing emerging data on the successful use of direct oral anticoagulants (DOACs) in managing HIT has been reported. An in vitro study showed that DOACs do not cause platelet activation or aggregation in the presence of HIT antibodies; the finding was attributed to the structural difference between DOACs and heparin products [5]. In a systematic review by Shatzel et al. of HIT treated with DOACs from January of 2012 to December 2015, the most commonly used DOAC was rivaroxaban (59%), followed by apixaban (28%), and dabigatran (13%) [6]. It is important to mention that 78% of all patients examined had prior treatment with non-heparin anticoagulant prior to initiation of DOACs. However, 22% of the 54 patients evaluated were treated with DOACs only. In this subgroup, there was one reported case of thrombus which eventually resolved with continued DOAC use; there was no reported HIT-related mortality [6]. A prospective single-arm study evaluating the use of rivaroxaban in patients with HIT was terminated due to poor recruitment [7], with generally good results observed in 12 patients with laboratory confirmed diagnosis of HIT. We report a new case of a patient with confirmed HIT following cardiovascular surgery treated with argatroban and successfully transitioned to apixaban.

## Case presentation

**Fig. 1:**
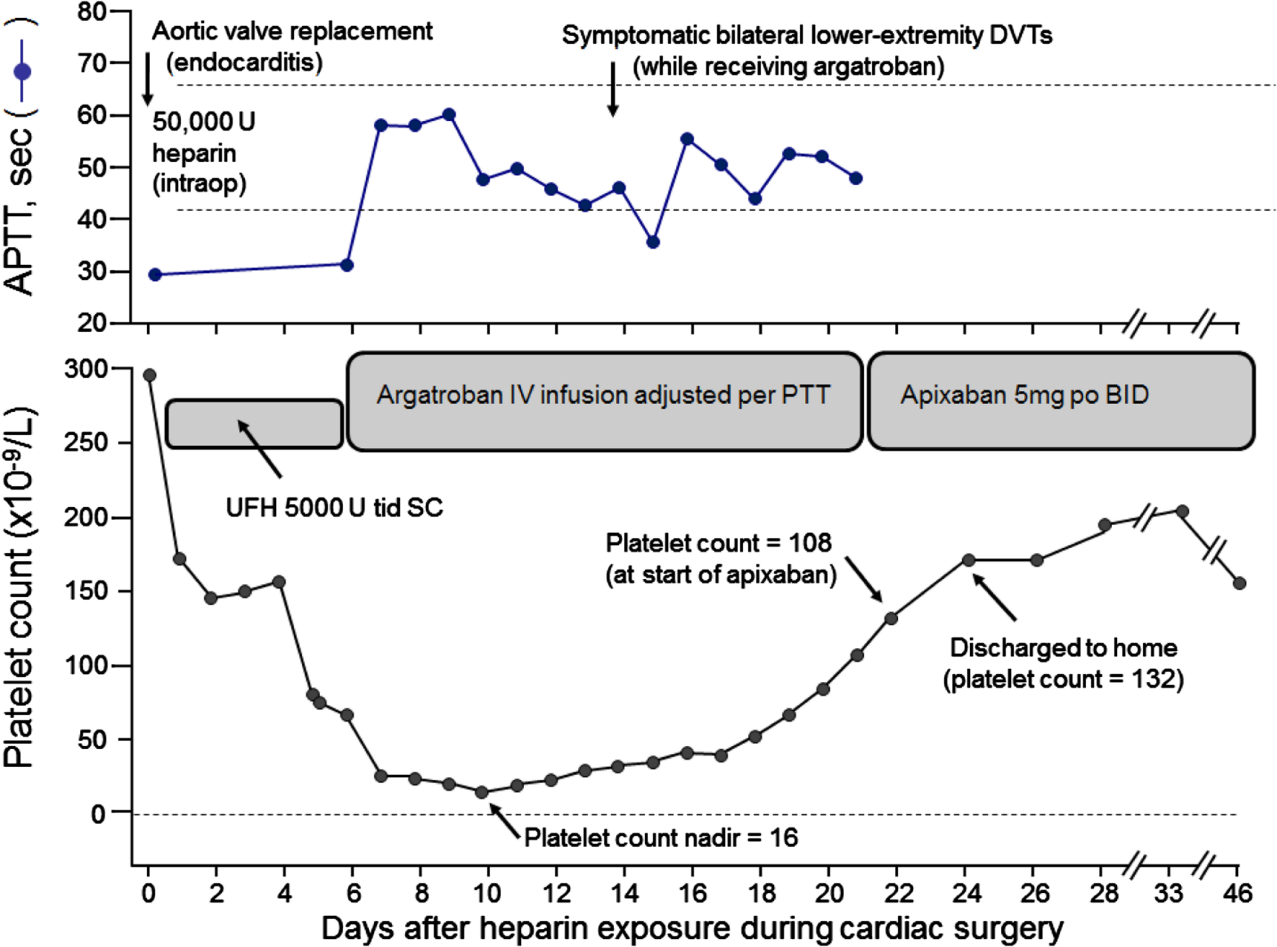
Schematic representation of activated partial thromboplastin time (APTT), platelet count and key anticoagulation management interventions following cardiovascular surgery. *BID* twice daily, *DVT* deep vein thrombosis, *po* by mouth, *PTT* partial thromboplastin time, *SC* subcutaneous administration, *tid* three times daily, *UFH* unfractionated heparin

A 66-year-old Caucasian male with chronic atrial fibrillation, aortic regurgitation, and hypertension presented to the emergency room with persisting fever, chills, fatigue and shortness of breath. Blood cultures were positive for Gram positive cocci in clusters (*Staphylococcus lugdunensis*), and he was started on appropriate antibiotics based on sensitivity results. Transesophageal 2D-ECHO revealed a mass on the aortic valve attached to the right coronary cusp causing prolapse of the cusp, findings highly suspicious for endocarditis. The patient subsequently underwent aortic root replacement with a Trifecta tissue valve and Gortex valsalva conduit. Intraoperatively, the patient received a loading dose of unfractionated heparin (40,000 Units) followed by 10,000 Units. The surgery was successful and the patient was started on routine post-operative antithrombotic prophylaxis with subcutaneous unfractionated heparin (5000 Units three times daily) starting on postoperative day (POD) 0. Following the expected early postoperative thrombocytopenia, the patient developed an unexpected further decline in the platelet count that began on POD 5, with further progression in thrombocytopenia resulting in an ultimate platelet count nadir of 16 × 10^9^/L on POD 10 (see Fig. 1). The patient had at least an intermediate 4Ts score for HIT based upon the timing and magnitude of platelet count fall and lack of alternative explanations for his thrombocytopenia. On POD 5, the heparin was stopped and argatroban started, with argatroban dosing adjusted based on routine laboratory values. An ELISA test for anti-multimolecular platelet factor 4 (PF4)/heparin antibodies was positive with a value of 1.131 optical density (OD) units (negative reference range 0.000–0.399) and confirmed with a strong positive C^14^-serotonin-release assay (SRA) [100% serotonin-release at low dose (0.1 IU/mL) of unfractionated heparin (UFH); and 0% at high-dose (100 IU/mL) UFH]. Interestingly, the patient’s serum also caused 100% serotonin-release at 0 IU/mL UFH (buffer control), which is a feature of autoimmune-like HIT antibodies that can explain thrombocytopenia that persists for several weeks despite stopping all heparin [8]. Despite prompt initiation of argatroban anticoagulation, the patient subsequently developed bilateral lower extremity edema on POD 14. Doppler ultrasound of both lower extremities was positive for right and left lower extremity deep vein thrombosis (DVT) (figure not shown), consistent with the known frequency of DVT in approximately 50% of patients with SRA-positive HIT [6].

The patient was continued on argatroban for 16 days with minimal improvement in platelet count (Fig. 1). At this time, the patient insisted on being discharged. Current guidelines on the management of HIT were reviewed with patient and family. In addition, we also discussed the few reported case series on the successful use of DOACs in the management of HIT, stressing clearly the limitations of using this drug including but not limited to lack of prospective, randomized studies, risk of publication bias and underreporting of failure incidence. The patient and family verbally confirmed understanding of the risks of using DOACs and requested discharge on an oral anticoagulant. Following a multi-disciplinary discussions, the patient was discharged on apixaban 5 mg orally twice daily. His platelet count was 108 × 10^9^/L when argatroban was discontinued and apixaban started immediately. This transitioning scheme is based on the half-life of argatroban which is 39–51 min in patients with normal hepatic function; of note, half-life may extend up to 181 min in patients with hepatic impairment, thus in such patients caution on appropriate transitioning protocol is recommended. Our patient was followed for >30 days with steady increase in platelet count, no new thrombosis (based on imaging, not shown) and no episodes of bleeding. The patient continues to follow with our clinic with no reported incidence of bleeding or thrombosis 3 months later.

## Discussion

HIT occurs in as many as 5% of patients exposed to heparin products, particularly patients who receive UFH for postoperative thromboprophylaxis for at least 1 week [9]. Although HIT also occurs with low-molecular-weight heparin (LMWH), the risk is approximately tenfold higher with UFH [10]. A meta-analysis by Martel et al. showed that even thromboprophylactic dosing of UFH and LMWH had an absolute risk of 2.6% (95% CI 1.5–3.8) and 0.2% (95% CI 0.1–0.4) respectively of producing HIT [11]. The observed risk of developing HIT is magnified in surgical (versus medical) patients [11]. A retrospective study of patients diagnosed with HIT between 2009 and 2011 revealed an increased rate of HIT in patients after cardiac surgery (0.53%, 95% CI 0.51–0.54), followed by vascular surgery (0.28%, 95% CI 0.28–0.29), and orthopedic surgery (0.05%, 95% CI 0.05–0.06) [12].

**Fig. 2:**
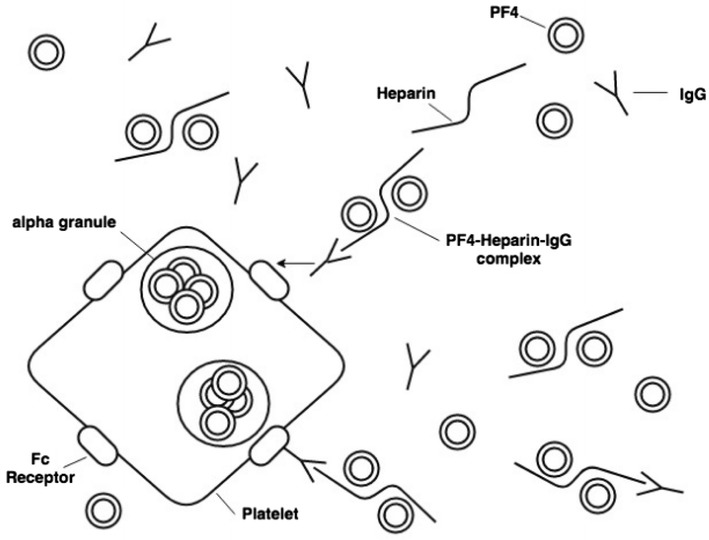
Mechanism of heparin-induced thrombocytopenia. Negatively charged heparin binds to positively charged platelet factor-4 (PF-4) forming antigenic multimeric complexes, resulting in IgG formation against these complexes. These antibodies bind to the Fc site on platelets and cause further PF-4 release from alpha-granules. Concurrently, monocytes are activated and release tissue factor, further activating thrombin and propagating endothelial damage

HIT has been categorized into two types. Type I is a benign, transient, non-immunologic condition that occurs early in heparin administration and is likely caused by the direct effect of heparin in causing platelet aggregation [13], and confers no increased risk of thrombosis [14]. In contrast, type II HIT is an immune-mediated, life and limb-threatening condition that begins 5 or more days following an immunizing exposure to heparin. The immune-mediated cascade is activated when (cationic) platelet factor 4 (PF4), released from platelet α-granules, interacts with (anionic) heparin, forming highly immunogenic multimeric PF4/heparin complexes. Resulting anti-PF4/heparin antibodies of IgG class bind to PF4/polyanion complexes formed in situ on platelet surfaces. These highly pathogenic IgG antibodies bind to FcɣIIa receptors, with Fc receptor complexing leading to strong platelet activation and aggregation [15], including the formation of procoagulant, platelet-derived microparticles (Fig. 2). Concurrently, monocytes are activated via their FcɣRI receptors, resulting in tissue factor production and further contributing to the procoagulant state. HIT thus is characterized by the paradox of a hypercoagulable state in the setting of thrombocytopenia, due to an immune-mediated disorder triggered by heparin administration. Ironically, the attempted prevention of thrombosis via heparin thromboprophylaxis results rather in an intense prothrombotic state that yields a risk of thrombosis of 12 to 15-times greater than in non-HIT controls [15].

Despite the fact that IgG isotypes remain the most commonly associated antibody, reports of only IgM and/or IgA has been documented. For instance, Amiral et al. showed that type II HIT can be induced by IgM and/or IgA anti-H-PF4 antibodies even in the absence of IgG antibodies [16]. They showed that there is increased expression of platelet PF4 receptors following a slight activation by thrombin which is subsequently followed by binding of IgM and/or IgA antibodies to H-PF4 on the surfaces of platelets. The sequela of this binding complex are platelet activation, aggregation and thrombosis. Apart from the above mentioned pathway for IgM and/or IgA induced thrombosis; other alternate pathways have been enumerated including (a) indirect effect of neutrophils, monocytes and lymphocytes on platelets resulting in thrombocytopenia and exposure of IgA and/or IgM receptors, (b) complement directed platelet activation or destruction via IgM-platelet complexes, (c) direct interaction between antibodies and glycosaminoglycans-PF4 complexes on endothelial surface resulting in increased procoagulant activities and thrombosis risk.

The implications of a misdiagnosis of HIT, either overdiagnosis or underrecognition—calls for keen clinical judgment and appropriating laboratory testing. Early diagnosis and treatment is essential in avoiding life or limb-threatening outcomes of HIT and equally necessary in circumventing unnecessary exposure of patients to direct thrombin inhibitors or other non-heparin anticoagulants, and their costs and risks.

**Fig. 3:**
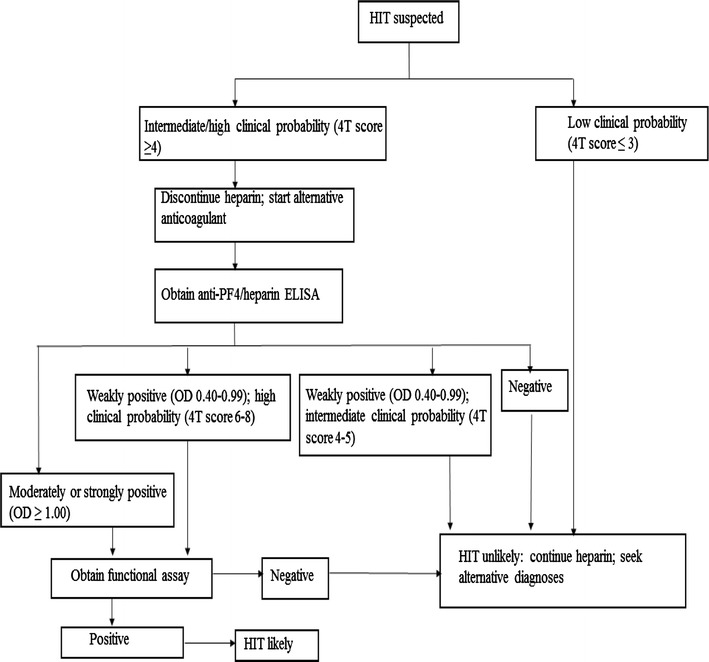
Diagnostic and treatment algorithm for suspected HIT. *HIT* heparin-induced thrombocytopenia, *OD* optical density (adapted from Cuker et al. [18])

Typically, the platelet count nadir in HIT is approximately 50–60 × 10^9^/L with only 10% of patients developing severe thrombocytopenia (≤15 × 10^9^/L) [17]. The fall in platelets usually begins within a 5–10 day window after an immunizing exposure to heparin, although it may occasionally take up to 14 days. Approximately 50–70% of patients with HIT develop associated thrombosis, most often venous (as seen in our patient) but also arterial in 10–15% of patients [18]. A scoring system aimed to help estimate the pretest probability of HIT, the 4Ts, assesses thrombocytopenia, Timing of onset (of thrombocytopenia and/or thrombosis) in relation to heparin use, presence of Thrombosis (or certain other characteristic sequelae of HIT), and exclusion of other etiologies that could explain the clinical course (Table 1). For a calculated score of at least intermediate risk (≥4 points), it is recommended that HIT management be initiated (Fig. 3).

**Table 1:** 4T’s, a pretest scoring system used in the clinical diagnosis of HIT. Adapted from Lo et al. [13]

Clinical feature	2 points	1 point	0 point
Thrombocytopenia	Platelet decrease >50% from baseline platelet nadir >20,000	Platelet decrease of 30–50% platelet nadir 10,000–19,000	Platelet decrease <30% platelet nadir <10,000
Timing of platelet decrease	5–10 days after heparin exposure OR ≤1 day (if exposed to heparin within 30 days)	Likely 5–10 days (incomplete evidence) OR onset after day 10 OR ≤1 day (if exposed to heparin within 30–100 days)	≤4 days without recent exposure
Thrombosis or other sequelae	New and confirmed thrombosis skin necrosis anaphylactic reaction to IV unfractionated heparin	Progressive/recurrent thrombosis non-necrotizing skin lesions unconfirmed thrombosis	None
Other etiologies for thrombocytopenia	None	Possible	Definite

High probability of HIT: 6–8 points; intermediate probability of HIT: 4–5 points; low probability of HIT: ≤3 points; recommended to discontinue heparin products and start non-heparin anticoagulant with score of ≥4; HIT: heparin-induced thrombocytopenia

Laboratory detection of HIT-associated antibodies is an important diagnostic endeavor. Although tests are institution-based, the most accessible test is a PF4-dependent ELISA, with high sensitivity (~99%) but limited specificity (<50%) [19]; thus, ELISAs when negative are an excellent tool to rule out HIT. In most cases, a positive ELISA warrants further evaluation with the more specific SRA (sensitivity and specificity >98 and 95% respectively) [19]. Our patient tested positive in both the ELISA and SRA. In our patient, the combination of a high clinical probability plus positive testing in both the immunoassay and a specific platelet activation assay, with the absence of any other plausible explanation for the clinical course, makes a diagnosis of HIT definitive.

Current practice for strong suspicion of HIT involves discontinuation of heparin products and prompt initiation of an alternative non-heparin anticoagulant, such as the direct thrombin inhibitor, argatroban. Argatroban is administered at a usual starting dose of 2 mcg/kg/min via continuous infusion. The dose is adjusted to goal APTT of 1.5–3.0 times patient’s baseline (with ~75% initial dose-reduction for patients with hepatic dysfunction, heart failure, post-cardiac surgery and anasarca) [20]. Bivalirudin is another direct thrombin inhibitor approved for managing the subset of HIT patients undergoing percutaneous coronary intervention [21]. Usually patients are continued on these non-heparin anticoagulants until recovery of platelet count (>150,000) before transitioning to warfarin. Both argatroban and bivalirudin require parenteral (intravenous) administration and frequent lab monitoring, ultimately resulting in longer hospital stay and expense for patients and hospitals.

**Fig. 4:**
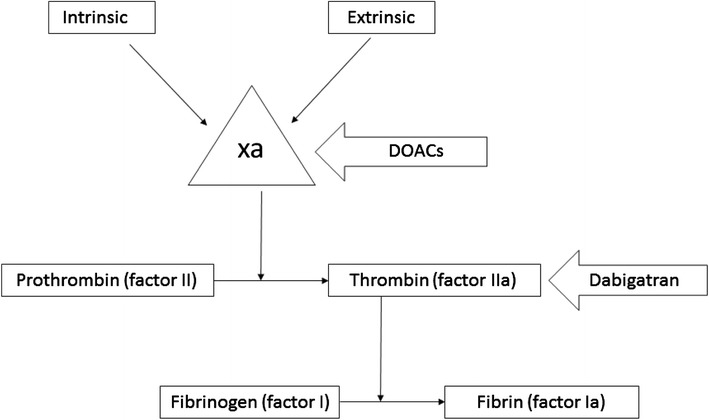
Schematic representation of the sites of action of direct oral anticoagulants and direct thrombin antagonists

Clinicians have examined alternatives to these drugs with the goal of cost-effectiveness while maintaining patient safety. Retrospective studies of the successful use of direct oral anticoagulants (DOACs) in managing suspected or confirmed HIT have emerged [4]. These direct factor Xa-inhibitors (Fig. 4) are currently approved for anticoagulation in non-valvular atrial fibrillation, treatment of thromboses (including pulmonary embolism and acute deep vein thrombosis) and thromboprophylaxis in postoperative patients. Oral factor Xa-inhibitors available in the USA include rivaroxaban, apixaban, and edoxaban. Dabigatran is an orally administered direct thrombin inhibitor (Fig. 4).

Apixaban is an oral direct anti-factor Xa inhibitor which has shown excellent anticoagulation characteristics and safety profile, and improved compliance when compared to most parentally administered anticoagulants [5]. The interaction of apixaban and platelet activity has been studied using HIT-positive sera, the C^14^-SRA platelet assay and the platelet aggregation assay. Apixaban did not react with HIT-antibodies, and platelets were not activated, evidenced by low levels of serotonin released by the platelets [5]. Sharifi et al., showed that 5 out of 22 patients with confirmed HIT initially treated with argatroban before transitioning to apixaban did well after more than 1 year follow up without any episodes of bleeding or thrombosis [22]. Based on our experience and other reported cases, we believe that a short course of argatroban followed by a DOAC is safe and effective in managing confirmed HIT; however, a randomized prospective study is needed to validate such claim. It is even possible that initial therapy with a DOAC, i.e., without initial therapy with a standard HIT anticoagulant such as argatroban, will be safe and effective therapy for patients with proven HIT.

## Conclusions

The advantages of using DOACs in the management of HIT are numerous, including increased patient compliance, decreased need for laboratory monitoring, fixed dosing and effective anticoagulation based on experience to date, even in the hypercoagulable milieu of acute HIT. DOAC administration is overall more convenient, poses a lower bleeding risk, eliminates warfarin-related skin necrosis and has fewer interactions between drugs and food compared to vitamin K antagonists [5]. In separate retrospective reports, successful use of DOACs in the treatment of confirmed HIT has been documented [9, 15, 23]. Other studies have also shown successful transitioning from direct thrombin inhibitors such as argatroban to DOACs in patients with confirmed HIT [22]. Our patient was safely transitioned to apixaban after 16 days of argatroban administration with excellent platelet recovery and no recorded thrombosis at 3-month follow-up.

## Abbreviations

HIT: heparin-induced thrombocytopenia
PF4: multimolecular platelet factor 4
SRA: C^14^-serotonin release assay
DOACs: direct oral anticoagulants
POD: post-operative day
DTI: direct thrombin inhibitor
UFH: unfractionated heparin
DVT: deep vein thrombosis
RLE: right lower extremity
LLE: left lower extremity
LMWH: low molecular weight heparin
OD: optical density
